# 5. Paediatric Takayasu arteritis

**DOI:** 10.1093/rap/rky031.004

**Published:** 2018-09-20

**Authors:** Madeleine Mackay, Ruud Verstegen, Daniel Hawley, Rachel Tattersall, Jenny Edgerton, Clare Nash, Anne-Marie McMahon

**Affiliations:** 1 Rheumatology, Sheffield Children's NHS Foundation Trust, Sheffield, UNITED KINGDOM; 2 Adolescent and Adult Rheumatology, Sheffield Teaching Hospitals NHS Foundation Trust, Sheffield, UNITED KINGDOM

**Introduction:** Takayasu arteritis is a chronic, granulomatous vasculitis of large vessels, involving the aorta and its major branches. Its aetiology is unknown and disease progression is variable with 10-year survival >90%. Takyasu arteritis is rare in children and young people (CYP). It is more frequent in Asian countries compared to Europe and North America, but the incidence is unknown. A 2010 review reported only 241 cases in CYP were reported worldwide. This case is of a 14 year old adolescent diagnosed with Takayasu arteritis six years ago aged eight. We outline the complex progression through his disease which has proved refractory to multiple treatments which themselves have caused morbidity. The case highlights many of the biomedical as well as psychosocial issues that can challenge teams looking after children and young people with rare conditions and the particular complexities that adolescence adds.

**Case description:** The patient was born in 2004 and grew up as part of a travelling family living on a static site. He had no health or developmental problems until 2012 when he presented with fatigue, weight loss, back pain, swollen ankles, and daily fever. At the time of presentation he had no arthritis, no skin rash, and no neurological signs. His left brachial pulse was diminished and right dorsalis pedis was absent (clinically and by USS). His left carotid pulse was present, but the right diminished (by USS). There was a difference of 20mmHg in systolic blood pressure in his arms (elevated on the right). His ESR was 108. A CT chest showed a widened mediastinum and renal USS showed stenosis of both renal arteries, more on the left side. An MRA showed diffuse thickening to the walls of both common carotids and proximal internal carotid bilaterally with 50% stenosis of the right internal carotid artery. Based on these findings, he was diagnosed with Takayasu arteritis. The patient was started on treatment with corticosteroids (initially intravenous later orally), intravenous cyclophosphamide and subcutaneous methotrexate. He was commenced on low molecular weight heparin (LMWH) with a plan to switch to aspirin at three months. His ESR improved to 22. The patient also started prophylactic voriconazole and valaciclovir. Once his cyclophosphamide was stopped, he continued to need weekly methylprednisolone in order to control symptoms and was therefore started on tocilizumab. It was also noted at this time that back pain was a herald on inflammation for the patient. In 2013, the patient had bilateral femoral head avascular necrosis and bisphosphonates were started. He was noted to have hypogammaglobulinaemia and was started on IVIG replacement under the immunology team. The patient was admitted with a two-month history of cough in September 2013. Bilateral cavitating lesions were seen on x-ray and critical airway narrowing made bronchoscopy difficult. Tuberculosis was excluded and he was treated for a fungal infection. Repeat chest imaging improved but showed a small pericardial effusion. He was difficult to wean off the prednisolone and remained on 20mg/day. Between November and December 2013, his pericardial effusion was monitored and gradually increased in size. Tocilizumab and methotrexate were restated once the fungal infection had resolved. In late December 2013, he developed tamponade and bilateral limb oedema and was transferred urgently to the nearest paediatric cardiology service. A pericardial drain was inserted and later a pericardial window was performed. The pericardial histology showed vasculitis. The tocilizumab was stopped and the patient started rituximab with cyclophosphamide 500mg for both initial treatment pulses. Between 2014 and 2015 the patient was noted to have elevated transaminases due to steatosis as well as problematic hypertension. Following cyclophosphamide, mycophenolate mofetil was added to his treatment. Rituximab and prednisone were continued. He continued to require IV methylprednisolone with episodic inflammation often heralded by back pain. A compression fracture L1 occurred. In November 2016, the patient presented with a three-week history of back pain and night sweats. MRA showed new carotid artery inflammation and dilation of the pulmonary arteries. He had a PET requested and a further review at Great Ormond Street Hospital (GOSH) suggested retrying tocilizumab and to anticoagulate with LMWH as well as aspirin until the inflammation was controlled. By January 2017, his symptoms had improved and the LMWH was stopped. In November 2017 the patient had continued back pain. He was given a trial of IV magnesium under care of the pain team and was started on pamidronate 1mg/kg. Due to persistent diarrhoea, the tocilizumab frequency was decreased to once every four weeks. In March 2018 he had a cardiology review. On echo, in addition to his known pulmonary artery dilatation, there was a dilated right proximal coronary artery and aortic sinus dilatation. Later that month he was admitted with back pain, hypertension and stridor, all of which settled with steroids, and headaches, which settled with control of blood pressure. An ultrasound of his carotids showed increased vessel wall thickness and inflammation when compared to the scan he had in November 2017. Renal Doppler also showed increased acceleration parameters suggesting renal stenosis compared to values from November 2017. An MRA of brain, chest, and abdomen showed evidence of disease progression when compared to scans from August 2017 and November 2016. There was aortic wall enhancement generally, with specific enhancement at renal artery origin and evidence of stenosis, a focal area of enhancement and stenosis at distal left common carotid at bifurcation and no neuroparenchymal abnormalities. The decision was made to increase the patient’s dose of tocilizumab to two-weekly and to give a pulse of IV methylprednisolone. Methotrexate was added, but stopped after two doses due to bone marrow suppression. The mycophenolate mofetil was stopped for the same reason. On follow-up imaging the inflammatory changes improved, but the renal artery stenosis remained for which he has been referred to GOSH. Throughout his treatment the patient and his family have been involved in discussions around treatment, but also prognosis. When he was admitted in 2016, a formal end of life discussion was held. The patient lives with an unpredictable risk of death, knowing that he has a potential of acute sudden collapse or stroke at any point. He has a strong faith, as do his family. Whenever he has acute pain there is high anxiety as to what is happening for him and a fear of procedures. He has requested to be present at all discussions, especially those regarding prognosis and end of life care. Only recently has he agreed to meet with our psychologist.

**Discussion:** Treatment decisions in this case have often been challenging, as paediatric Takayasu arteritis is a rare condition with sparse evidence to guide practice. Decisions have often been made with consultation of clinicians across the country and by extrapolating principles from adult rheumatology practice. Steroids remain the mainstay for the patient’s Takayasu arteritis, in combination with tocilizumab. The treatment goals remain to control the disease while minimizing side effects of treatment to improve his quality of life. There has been continued care planning, including transition planning from paediatric to adult services. Depending upon the progression of his disease, end of life care may need to be re-discussed.

**Key Learning Points**: Treatment of Takayasu arteritis in children and young people is challenging and requires a collaborative approach with experts around the country, and occasionally around the world. Burden of the disease is not restricted to the physical symptoms, but also the treatment-associated morbidity and the psychological and social problems associated with chronic, refractory illness in CYP. The disease not only affects the young person, but the family too and psychological support should be offered; although access to psychology is often severely restricted. CYP need to be involved in discussions about their disease, its treatment and end of life care. This is particularly important for adolescents.

**Disclosure: M. Mackay:** None. **R. Verstegen:** None. **D. Hawley:** None. **R. Tattersall:** None. **J. Edgerton:** None. **C. Nash:** None. **A. McMahon:** None.

